# Bart syndrome: A case report of neonatal disorder

**DOI:** 10.1002/ccr3.8528

**Published:** 2024-02-09

**Authors:** Mohammad Amin Eghtedari, Monireh sharafi, Abes Ahmadijazi

**Affiliations:** ^1^ Student Research Committee Dezful University of Medical Sciences Dezful Iran; ^2^ Ali‐Asghar children's hospital, School of Medicine Iran University of medical sciences (IUMS) Tehran Iran; ^3^ Department of pediatrics and neonatology Dezful University of Medical Sciences Dezful Iran

**Keywords:** aplasia cutis, ectodermal dysplasia, epidermolysis bullosa, infant

## Abstract

Bart Syndrome, characterized by congenital skin absence, blistering, and nail abnormalities, presents complex neonatal challenges. This rare condition demands a multidisciplinary approach for accurate diagnosis and comprehensive care.

## 1 INTRODUCTION

A medical condition characterized by the congenital absence of skin, predominantly affecting the lower extremities, blisters on both skin and mucous membranes, and congenital absence and deformities in nails, has been identified within a familial context.^1^ The inheritance pattern aligns with a fully penetrant, autosomal dominant gene.^1^

In 1966, Bart introduced Bart syndrome, a condition marked by the simultaneous occurrence of congenital epidermolysis bullosa, localized congenital absence of skin affecting the extremities, and the abnormal shedding or dystrophy of nails.^2^ This syndrome is fascinating in the medical field due to its more optimistic prognosis when contrasted with other epidermolysis bullosa types.^2^ This type is known as Aplasia cutis congenital type VI.^3^

This syndrome is classified as an autosomal dominant disorder, with symptoms typically manifesting on the limbs, extending medially from distal regions. The manifestation includes the appearance of shiny, red lesions. The disease predominantly occurs in areas of the body that experience higher friction.^4^

While various manifestations exist in this disease, more severe cases of Bart's syndrome, particularly those associated with epidermolysis bullosa, may involve other congenital anomalies. These can include pyloric atresia, ureteral stenosis, renal abnormalities, underdeveloped ears, a flattened nose, a broad nasal root, and wide‐set eyes, all of which are observable in these patients. According to studies, there is no known specific underlying cause for Bart's syndrome, which is why both its etiology and pathophysiology are subjects of extensive debate.^5^

The diagnosis of Bart's syndrome is typically based on the patient's clinical symptoms, with a confirmatory diagnosis often achieved through skin biopsy. Additionally, genetic studies to identify gene mutations can be instrumental in confirming the final diagnosis.^5^

In this study, we have presented a case of a neonate with some disorders that guide us to syndromes, like Barth syndrome, that have contributed.

## 2 CASE PRESENTATION

### 2.1 Case history

In October 2023, a premature baby was born at Ganjavian Hospital of Dezfull. His arrival was not without complexities, as he brought a puzzling array of skin symptoms and multiorgan anomalies. This baby was born after 33 weeks of pregnancy to a 24‐year‐old Iranian mother experiencing her first pregnancy. The hospital's gynecological surgery team performed the cesarean delivery. As the baby took his first breaths, the attending medical staff noted an initial Apgar score of 8/10, which swiftly improved to a reassuring 9/10 within the first 5 min.

Within the context of the baby's family history, a significant detail emerged—the parents were closely related by blood, and there were no known instances of similar anomalies within their respective families. The decision to opt for a cesarean section was motivated by the presence of meconium in the baby's amniotic fluid, a precautionary measure taken to protect his well‐being. Regarding physical stature, both parents fell comfortably within the normal range—standing tall at approximately 180 cm with a weight of 80 kg for the father and a height of 168 cm with a weight of 64 kg for the mother. Furthermore, the mother's blood type was identified as A+.

**FIGURE 1:**
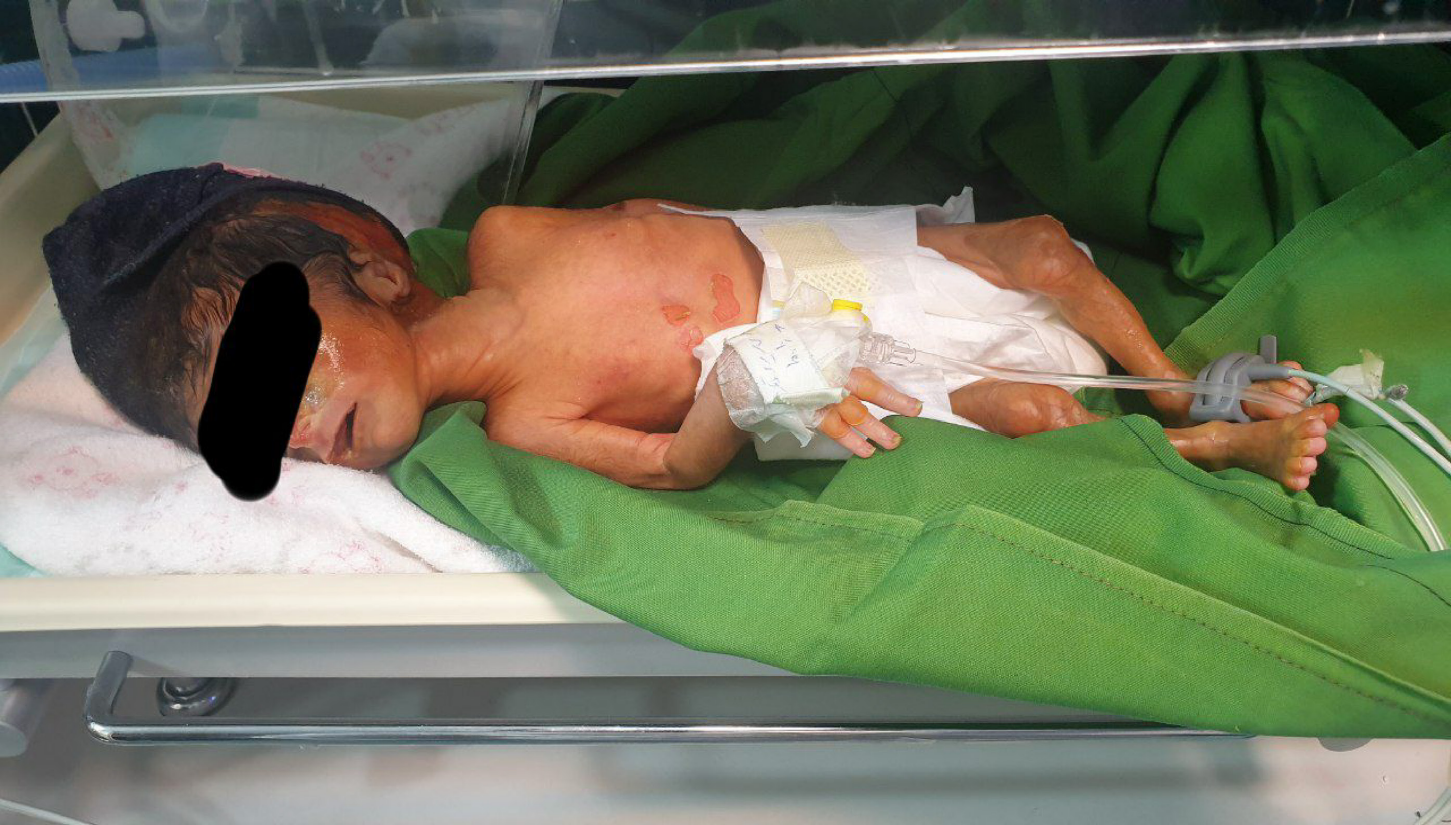
Overall patient condition.

Upon his arrival, the baby weighed a delicate 1500 gr and measured 42 cm in length, with a head circumference of 31.5 cm. The temperature at birth held steady at 36.7C while vital signs were meticulously recorded—his heart rhythm was 140 beats per minute, and his respiratory rate was 68 breaths per minute (Figure 1). From the moment of birth until the time of this composition, spanning approximately 4 days, the baby has been under the dedicated care of the Neonatal Intensive Care Unit (NICU). During this period, he has displayed pronounced intercostal retractions, the poignant sound of grunting, and the telltale sign of nasal flaring. A thorough examination at birth also unveiled an open rectum, and apnea episodes further added to the complexity of his condition.

Despite these challenges, it is noteworthy that the baby was not macrosomic, nor did he suffer from intrauterine growth restriction (IUGR). Hearteningly, there were no indications of deceleration or irregular heart rate patterns, and premature rupture of membranes (PROM) was not factored into this medical narrative.

### 2.2 Methods

**FIGURE 2:**
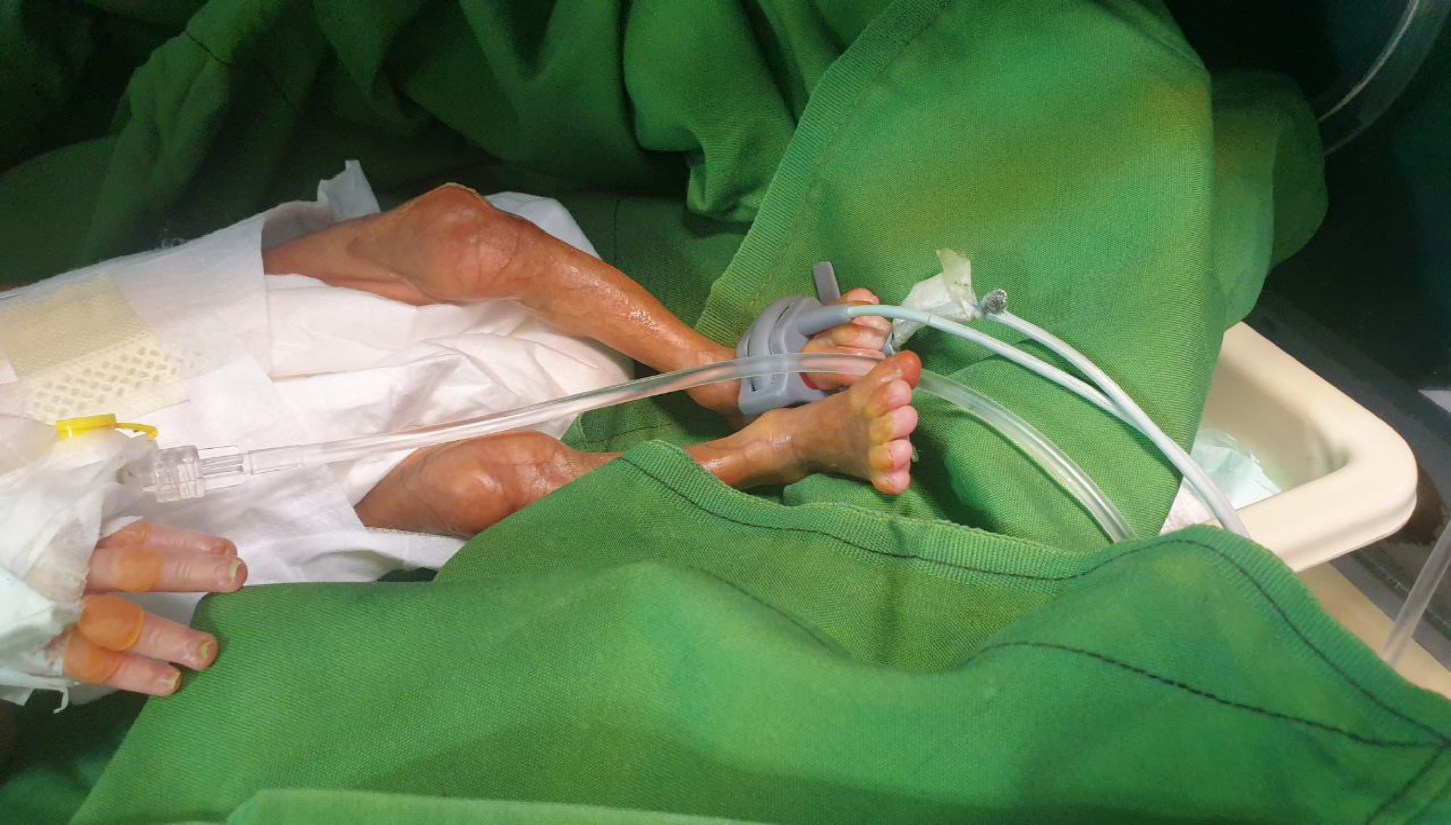
Dryness and inadequate formation of skin on the limb.

**FIGURE 3:**
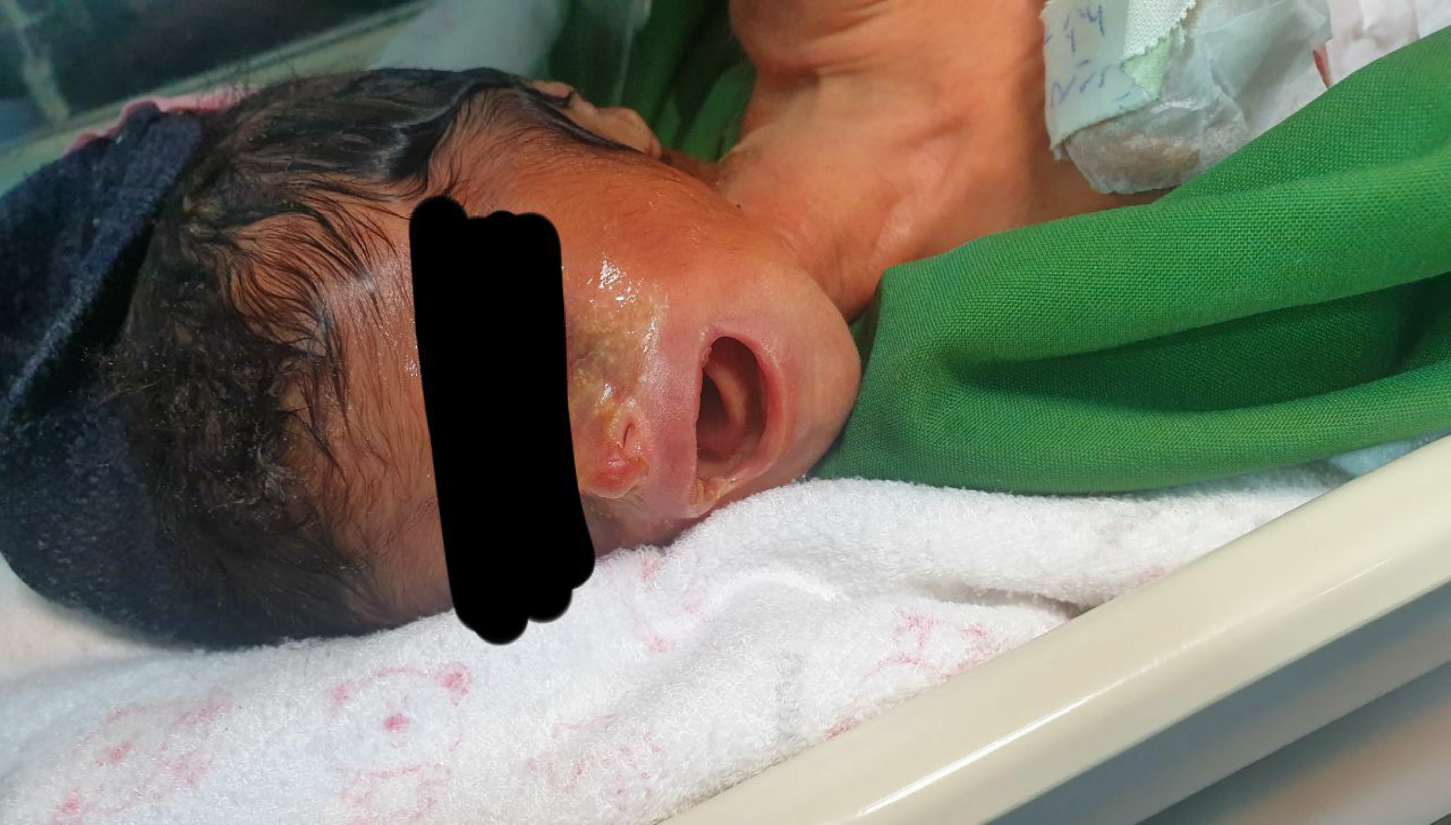
Skin abnormalities and ear and nose abnormalities.

As our gaze approaches the baby's delicate skin, we encounter a mosaic of anomalies—ecchymosis, skin breakdown, and even the absence of skin formation in specific areas (Figure 2). These areas encompass the precious head, expressive face, tender neck, left hand, dainty feet, and even the genitalia. The baby's left earlobe reveals aplasia and an entire formation of the right ear is regrettably absent. Additionally, aplasia extends into the nasal region, further underscoring the intricate nature of this medical tapestry (Figure 3).

During the initial assessment of the baby, we encountered a concerning array of symptoms related to their eyes. The corneal cloudiness and the strikingly white appearance of the pupils immediately caught our attention, pointing to the presence of corneal opacities and an unusually smooth corneal surface. Recognizing the gravity of this situation, we promptly requested a consultation with an ophthalmologist to evaluate the baby's ocular health thoroughly.

The subsequent eye examination confirmed the presence of corneal cloudiness, but it was intriguingly limited to the central cornea, with the periphery maintaining a reassuringly normal appearance. This finding led the medical team to conclude that the corneal opacity fell within an acceptable range of variation. It is worth noting that the baby displayed an unusual inability to keep their eyelids open, a phenomenon often associated with underlying issues. However, notably absent was any sign of ectropion, the outward turning of the eyelids, which was a positive indication.

Further investigations considered the presence of clubbing, the thickening of the fingertips, initially observed during the baby's examination. After careful consideration and diagnostic assessment, the possibility of KID syndrome was introduced. In response to this potential diagnosis, a treatment regimen was established, comprising the administration of erythromycin eye ointment twice daily to address the corneal issues and a weekly application of lubricating ointment to alleviate ocular discomfort.

**FIGURE 4:**
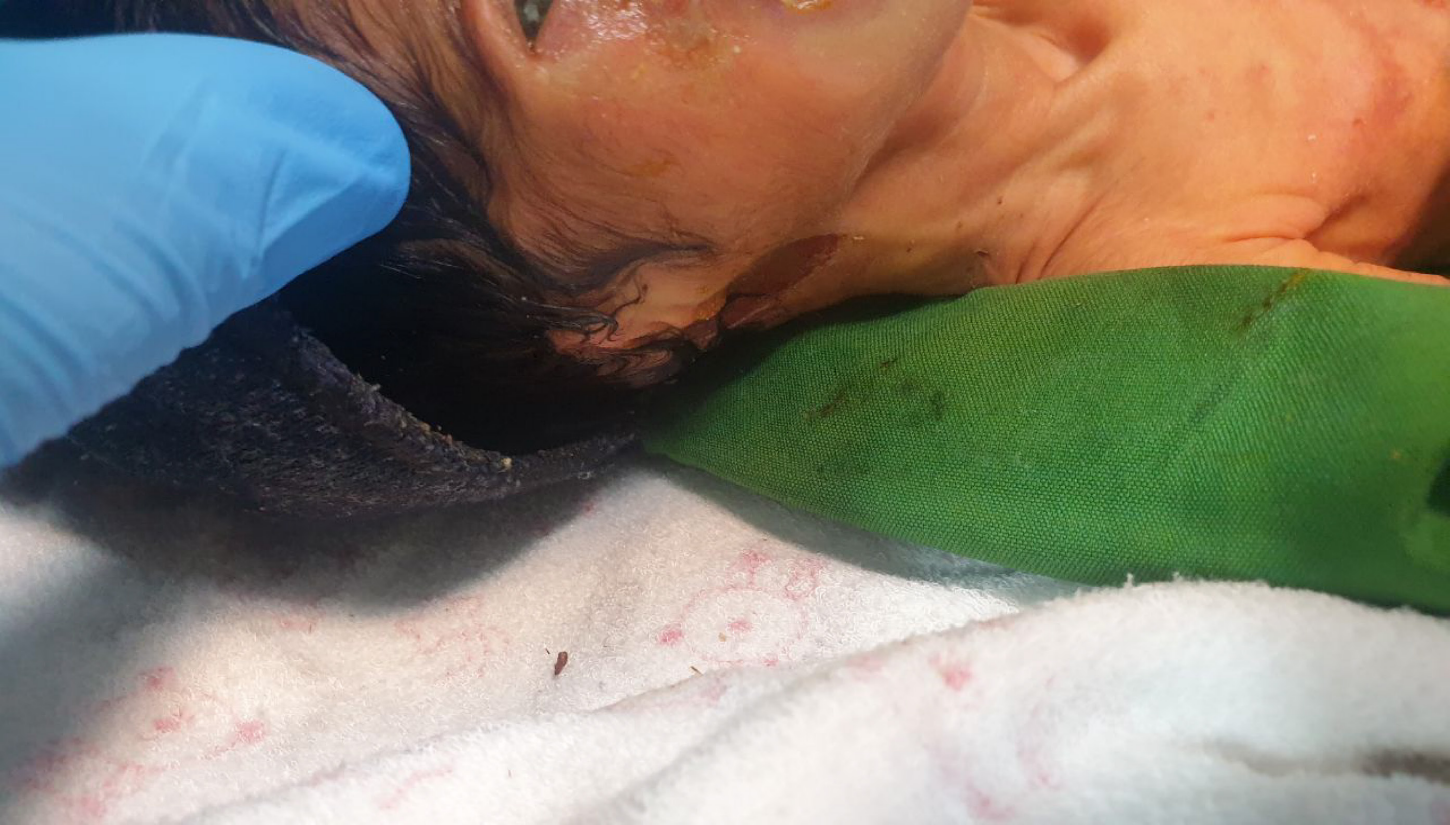
Inappropriate conditions of the right ear.

In dermatological evaluations, our scrutiny extended to the baby's delicate skin. The findings revealed disseminated vesicles and skin ecchymoses, forming a pattern indicative of dermatolysis. During this examination, an alarming discovery was made—a lack of reflex to light, an observation that raised concerns about possible visual impairment, particularly in light of the ear anomalies in the baby's medical profile (Figure 4).

The baby presented us with several additional challenges in the broader context of medical examinations. Notably, we could not detect audible heart sounds during our assessment, and when they were faintly present, it raised potential red flags regarding cardiac issues. Additionally, the baby exhibited bilateral cryptorchidism, a condition characterized by undescended testes, and we observed early separation of the umbilical cord, which warranted close monitoring. Encouragingly, the results of the blood culture and C‐reactive protein (CRP) levels fell within the normal range, offering a glimmer of reassurance amid this complex medical journey (Tables 1,2).

**TABLE 1:** laboratory findings.

Laboratory					
BS: 73	MCH: 37.14	RBC: 4.47	MCV: 106.4	Lymph: 51.3	NRBC: 70
Ca: 8.8	MCHC: 35.2	HB: 16.6	Poly: 40	MPV: 9.3	Mono: 5.5
WBC: 11.6	RDW: 11	Hct: 47.4	EOS: 3	Plt: 319	

**TABLE 2:** ABG findings.

ABG of the umbilical cord	
pH: 7.374	pO_2_: 34.9
pCO_2_: 45.3	Hct49
After 3 h	
pH:7.29	pO_2_: 55.2
pCO_2_: 37.5	

**FIGURE 5:**
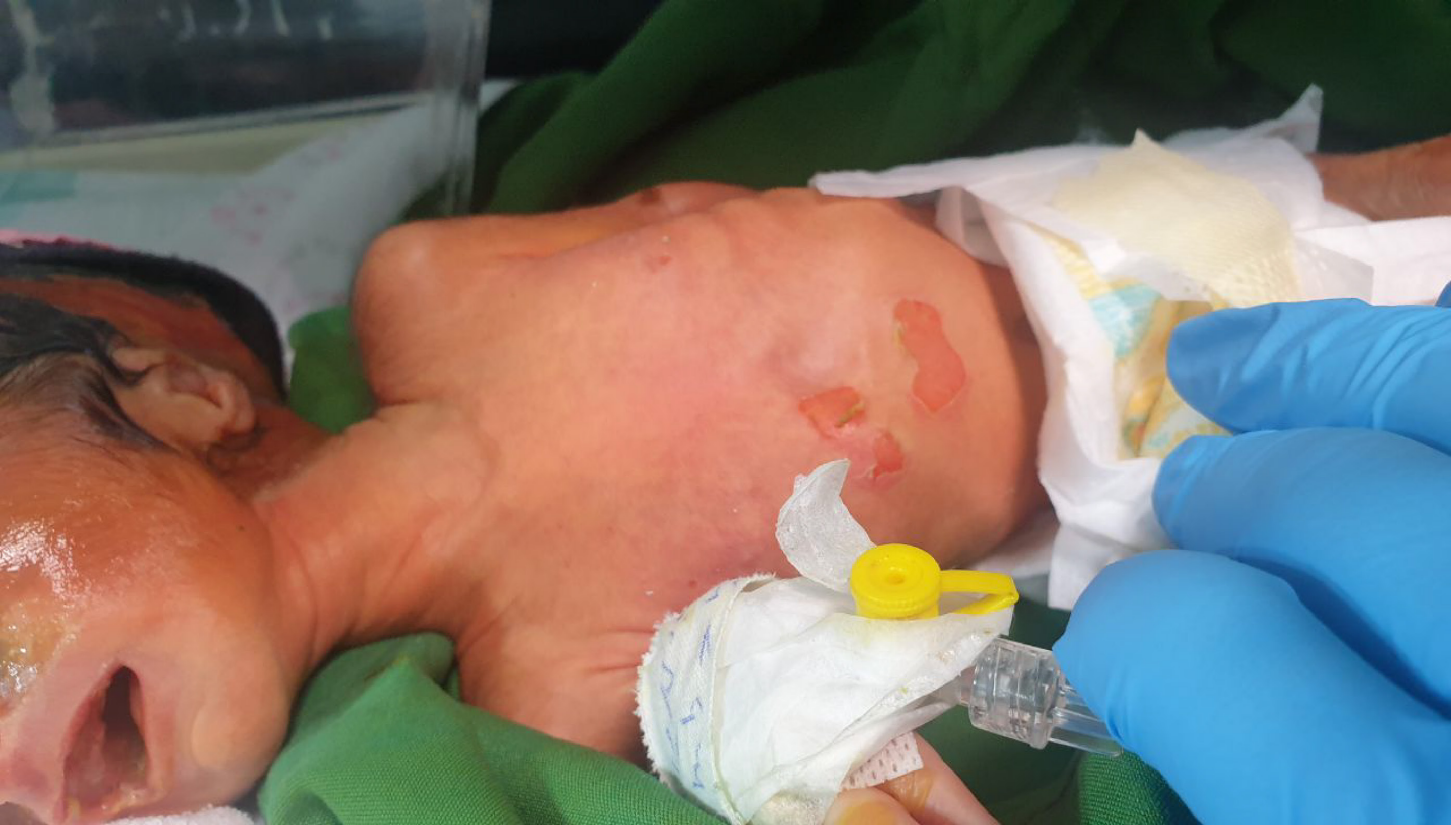
Skin surface erythemas.

**FIGURE 6:**
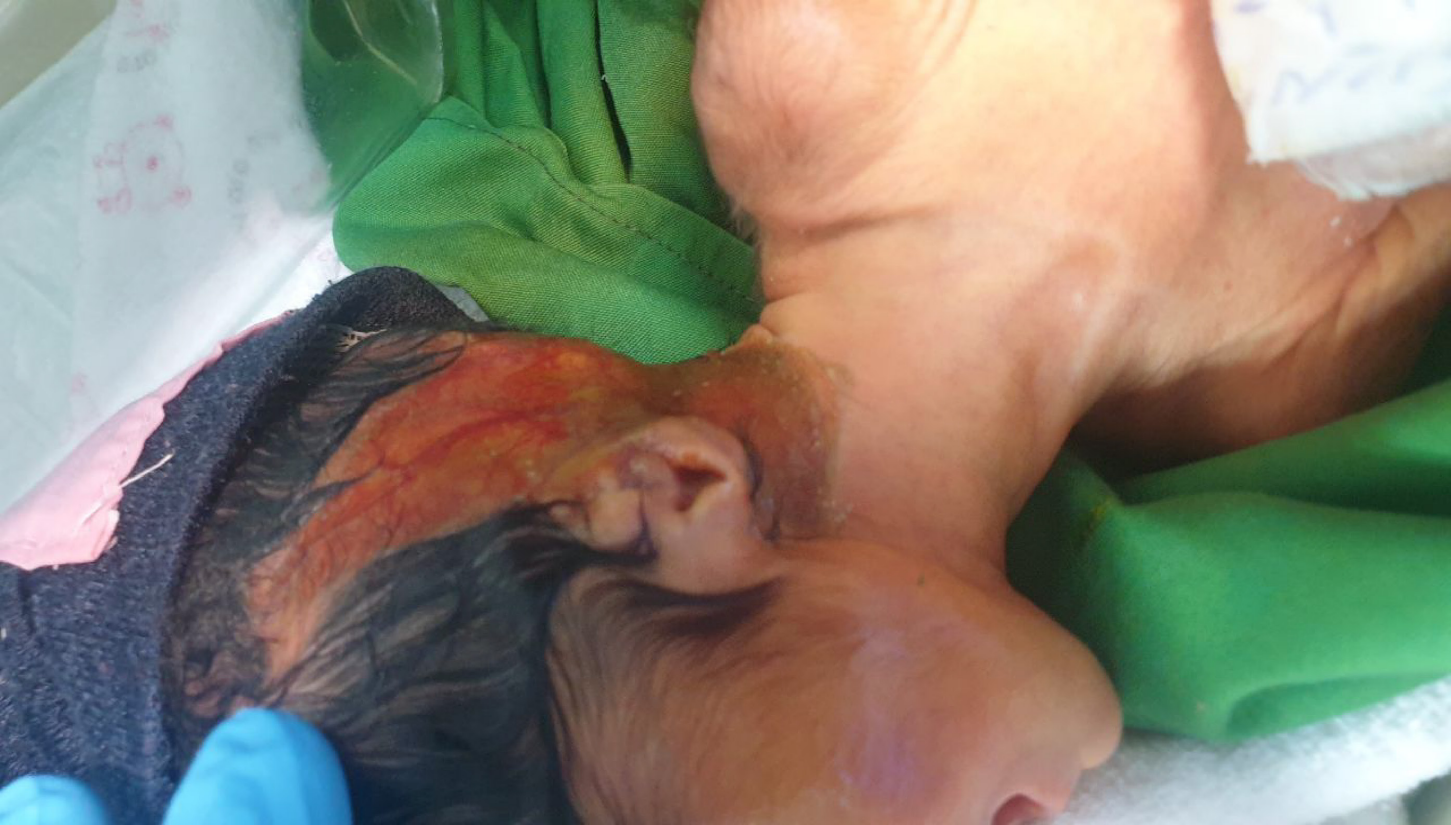
Separation and inadequate formation of scalp skin due to dryness and illness.

To manage the respiratory distress, skin issues, visual impairments, and mental challenges presented by the newborn, the decision was made to admit the baby to the Neonatal Intensive Care Unit (NICU) for specialized care (Figures 5,6). The baby was placed in a temperature‐controlled incubator within this unit to provide the optimal environment for their recovery and well‐being.

A comprehensive regimen of medications and therapies was established for the baby's treatment. To alleviate the breathing difficulties, the baby was administered gentamicin and ampicillin intramuscularly to combat potential infections. The skin conditions were treated with a combination of topical medications, including an emulsion of RDen, mupirocin ointment, Lubratax ointment, erythromycin ointment, and gentamicin eye ointment. These treatments aim to alleviate skin issues and promote healing.

In addition to these measures, meticulous eye care was essential due to visual impairments. Gentamicin eye ointment and injections managed potential eye infections and protected the baby's delicate eyesight.

Furthermore, a portable brain ultrasound on the newborn revealed no evident signs of hydrocephalus or midline shift, providing some relief from concerns related to these conditions. There were no indications of intraventricular hemorrhage (IVH) or cerebral parenchymal lesions, underscoring the importance of early monitoring and intervention to safeguard the baby's neurological health. The multidisciplinary approach in the NICU, combining respiratory support, dermatological care, eye treatments, and neurological assessments, aimed to provide the newborn with the best possible care and enhance their prospects for a healthy future. Close monitoring and tailored interventions will continue to be essential to the baby's ongoing care and treatment plan. Generally, care strategies for this disease are primarily confined to supportive measures and the monitoring of the patient's vital signs.

Despite all efforts for further investigations and actions to treat the patient, the baby's family finally discharged the baby from the hospital with personal consent.

## 3 CONCLUSION AND RESULTS

The mature epidermis is a stratified epithelial tissue composed predominantly of keratinocytes.^6^ A syndrome, as related to genetics, is a group of traits or Conditions that tend to occur together and characterize a recognizable disease. Some syndromes have a genetic cause.^7^

Bart syndrome is an unusual condition that combines features from two distinct disorders, namely aplasia cutis congenita (ACC) and epidermolysis bullosa (EB).^8^ ACC, a rare congenital anomaly characterized by the absence of skin, was initially documented in 1767 by Cordon.^8^ Frieden's classification system categorizes ACC into nine groups based on location and associated abnormalities.^8^

Inherited EB is a group of genetically transmitted skin disorders that can be part of other syndromes.^9^ It can be marked by skin and mucous membrane fragility, resulting in blisters with minimal trauma.^10^ The clinical diagnosis of aplasia cutis congenital, specifically Bart syndrome, hinges primarily on identifying classical cutaneous manifestations, with the extent of involvement contingent upon the mode of inheritance.^11^

In alignment with Omran et al. and Sharif, our case also exhibited skin lesions and ear malformation.^3^ In this case, we have seen standard Apgar scores like Sharif et al.^3^ It could be better to have a skin biopsy and Brain MRI. To achieve a precise postnatal classification of inherited Epidermolysis Bullosa (EB), it is imperative to employ skin biopsy as a fundamental diagnostic tool.^11^ This biopsy specimen should undergo comprehensive examination, incorporating a blend of ultrastructural and antigenic assessments through transmission electron microscopy, immunofluorescence antigenic mapping, and investigations utilizing EB‐specific monoclonal antibodies.^11^ In the treatment of this child, due to a skin disorder, the use of CPAP was not possible. Additionally, vascular access was not performed, and an umbilical catheter was placed. Antibiotics were administered due to suspicion of a skin infection with Staphylococcus Aureus, and the treatment was carried out with ampicillin and amikacin.

Management of Bart syndrome involves a comprehensive approach, including conservative measures, secondary intention healing, and surgical interventions when warranted. Traditional care primarily focuses on localized wound treatment and infection control using systemic antibiotics.^8^ The routine administration of systemic antibiotics is not a standard practice, but they may be considered if there are concerns about infection. While conservative methods suffice for most patients, those with substantial or deep wounds may require surgical interventions such as skin grafting or local flap procedures.^8^, ^12^

## 4 DISCUSSION

Bart syndrome, an infrequent congenital skin disorder characterized by its distinct clinical features, emphasizes the importance of prompt and conservative management in optimizing outcomes. Vigilant patient monitoring is advised for tracking progress. The first examination of newborns can guide us to critical situations. Some signs are straight to the diagnosis, but others are conflicting, and one should search for another disorder already in syndromes, so physicians should be aware of many syndromes and associations so as not to miss the exact diagnosis. Given the absence of accurate data on the prevalence of this disease, there is a noticeable need for comprehensive studies aimed at precisely evaluating its incidence. In relation to this disease, despite its poor prognosis, it is recommended to expedite all diagnostic tests, including skin biopsy, to ensure a definitive diagnosis. Concurrently, it is crucial to implement supportive care measures for the infant. Continuous monitoring of the infant's vital signs is essential to effectively manage their condition.

## AUTHOR CONTRIBUTIONS

**Mohammad Amin Eghtedari:** Writing – original draft; writing – review and editing. **Monireh Sharafi:** Writing – review and editing. **Abes Ahmadijazi:** Project administration; supervision; writing – review and editing.

## FUNDING INFORMATION

The expenses associated with this research have been covered by the authors, and financial support has been provided by the researcher.

## CONFLICT OF INTEREST STATEMENT

The authors declare no conflicts of interest related to the research presented in this article. There are no financial or personal relationships with individuals or organizations that could inappropriately influence or bias the content and findings of this work.

## ETHICS STATEMENT

Informed consent was obtained from participants in the study after explaining the nature and possible consequences of the study. This information has been taken from the parents of the patient the confidentiality of participant information has been strictly maintained, and any potential risks have been minimized to the best extent possible. For further information regarding this research's ethical aspects, please contact the author.

## CONSENT

Written informed consent was obtained from the patient to publish this report in accordance with the journal's patient consent policy.

## Data Availability

The data that support the findings of this study are available in the Warehouse of medical documents of Ganjavian Hospital. For inquiries regarding the data, please contact Mohammad Amin Eghtedari at amin.eghte@gmail.com.
